# Advancing Diagnostics with Semi-Automatic Tear Meniscus Central Area Measurement for Aqueous Deficient Dry Eye Discrimination

**DOI:** 10.3390/medicina61081322

**Published:** 2025-07-22

**Authors:** Hugo Pena-Verdeal, Jacobo Garcia-Queiruga, Belen Sabucedo-Villamarin, Carlos Garcia-Resua, Maria J. Giraldez, Eva Yebra-Pimentel

**Affiliations:** 1 GI-2092 Optometry, Departamento de Física Aplicada (Área de Optometría), Universidade de Santiago de Compostela, 15782 Santiago de Compostela, Spain; hugo.pena.verdeal@usc.es (H.P.-V.); belen.sabucedo@rai.usc.es (B.S.-V.); carlos.garcia.resua@usc.es (C.G.-R.);; 2 AC-24 Optometry, Instituto de Investigación Sanitaria de Santiago de Compostela (IDIS), 15706 Santiago de Compostela, Spain

**Keywords:** tear meniscus central area, aqueous deficient dry eye, tear film, diagnostic method

## Abstract

*Background and Objectives*: To clinically validate a semi-automatic measurement of Tear Meniscus Central Area (TMCA) to differentiate between Non-Aqueous Deficient Dry Eye (Non-ADDE) and Aqueous Deficient Dry Eye (ADDE) patients. *Materials and Methods:* 120 volunteer participants were included in the study. Following TFOS DEWS II diagnostic criteria, a battery of tests was conducted for dry eye diagnosis: Ocular Surface Disease Index questionnaire, tear film osmolarity, tear film break-up time, and corneal staining. Additionally, lower tear meniscus videos were captured with Tearscope illumination and, separately, with fluorescein using slit-lamp blue light and a yellow filter. Tear meniscus height was measured from Tearscope videos to differentiate Non-ADDE from ADDE participants, while TMCA was obtained from fluorescein videos. Both parameters were analyzed using the open-source software NIH ImageJ. *Results:* Receiver Operating Characteristics analysis showed that semi-automatic TMCA evaluation had significant diagnostic capability to differentiate between Non-ADDE and ADDE participants, with an optimal cut-off value to differentiate between the two groups of 54.62 mm^2^ (Area Under the Curve = 0.714 ± 0.051, *p* < 0.001; specificity: 71.7%; sensitivity: 68.9%). *Conclusions:* The semi-automatic TMCA evaluation showed preliminary valuable results as a diagnostic tool for distinguishing between ADDE and Non-ADDE individuals.

## 1. Introduction

Tears are produced by the lacrimal system and spread across the ocular surface during blinking, leaving primarily through evaporation and drainage [1]. The inferior lacrimal meniscus plays a key role in ocular surface health, contributing significantly to the stability and function of the tear film [2,3]. Its evaluation can offer indirect information about total tear volume on the ocular surface [2,3]. A thorough evaluation of its state is imperative for obtaining insights into ocular health and addressing disorders associated with this vital structure. Accordingly, to the Tear Film Ocular Surface second Dry Eye Workshop (TFOS DEWS II), one of the main disorders that could be assessed with this evaluation is Dry Eye Disease (DED), which was defined as “*a multifactorial disease of the tears and ocular surface that results in symptoms of discomfort, visual disturbance, and tear film instability with potential damage to the ocular surface. It is accompanied by increased osmolarity of the tear film and inflammation of the ocular surface*” [4]. Although the present study was designed and conducted according to the TFOS DEWS II criteria [4], the recent publication of the TFOS DEWS III report (2025) provides an updated, though largely consistent, framework for the definition and classification of DED [5]; this revised diagnostic model may guide future research in this area. Nevertheless, DED is influenced by factors other than the reduced volume of tears on the ocular surface. It can manifest in various forms, including Aqueous Deficient Dry Eye (ADDE), Evaporative Dry Eye (EDE), or a combination of both, known as MixedDED [4,6,7]. It is therefore vital that the evaluation of tear meniscus characteristics is incorporated into a routine clinical protocol for diagnosing and classifying DED, with a view to facilitating the early detection of potential ADDE cases [8,9,10,11,12].

Tear meniscus characteristics are commonly measured at the central portion of the lower eyelid, particularly tear meniscus height [12,13], or in multiple positions (nasal, central, and temporal) to assess regularity [14]. Recent technology advancements have led manufacturers to launch different instruments on the market. On one hand, there are the multi-diagnostic devices that have multiple measurement capabilities, such as the Dry Eye option for measuring tear meniscus height manually [15]. On the other hand, new devices with high-resolution imaging technologies exist, such as Optical Coherence Tomography (OCT) [9,10,12,15,16]. However, these measurements present two main shortcomings that may limit their clinical applicability and accuracy. First, the evaluation based solely on the height at a single measurement point may not adequately capture the overall morphology or functional status of the entire meniscus, potentially overlooking regional variations or localized abnormalities. Second, performing a more comprehensive and detailed analysis that encompasses the full extension and structure of the meniscus often necessitates the use of sophisticated, high-cost, and specialized imaging equipment, which may not be readily available in many clinical or research settings. To overcome these limitations, software-assisted image processing has been increasingly used [17,18]. The free-domain software ImageJ is a tool that aids in the quantification of features viewed in a picture and is used in the present study for meniscus quantification [19,20]. This software facilitates measurement and may enhance the resolution and repeatability when meniscus parameters are measured [21]. The study aimed to clinically validate a semi-automatic method to quantify the Tear Meniscus Central Area (TMCA), with the objective of distinguishing between participants with ADDE and those with Non-ADDE.

## 2. Materials and Methods

### 2.1. Participants and Study Design

This study is a cross-sectional observational analysis focused on participants diagnosed with ADDE. The sample size for the study was determined based on the TFOS DEWS II Diagnostic Methodology report, which recommends specific diagnostic tests [6]. The calculation was performed using the PS Power and Sample Size Calculations software, Version 3.1.2 (Copyright© by William D. Dupont and Walton D. Plummer) [22]. Assumptions were made for standard deviations (SDs) reported in the literature for key parameters: Ocular Surface Disease Index (OSDI) score (6.7), tear film osmolarity (4.8 mOsm/L), tear film break-up time (BUT) (2.9 s), and fluorescein corneal staining (2) [6,17,23,24,25]. To achieve 80% power (Type II error) at a significance level of α = 0.05 (Type I error) with a 2% margin of error, and to detect clinical differences between participants with and without DED of 7.3, 5 mOsm/L, 5 s, and 1, respectively, the minimum required sample sizes per group were 116, 88, 72, and 102, respectively, assuming a 1:1 control-to-experimental ratio. The highest of these values (116 participants) was selected to ensure study reliability.

A total of 120 participants with a DED diagnosis were included in the current study. All participants were referred to the optometry clinic by their medical practitioners for an ocular surface evaluation. The TFOS DEWS II Diagnostic Methodology Subcommittee criteria were employed to administer a series of tests to verify the diagnosis, including the OSDI questionnaire, tear film osmolarity, tear film BUT, and corneal staining [6,7]. Additionally, tear meniscus height (TMH) was assessed to classify the participants [12]. Furthermore, a video capturing the lower tear meniscus post-fluorescein instillation was recorded to obtain TCMA. All procedures were conducted by the same observer, who remained blinded to the questionnaire results. Subsequently, data were anonymized with an alphanumeric code for subsequent analysis.

Before the inclusion in the study, informed consent was obtained from all participants. The study underwent review and approval by the independent Bioethics Committee, ensuring adherence to ethical guidelines for the protection of human subjects in biomedical research.

### 2.2. Diagnostic and Classification Procedures and Criteria

Only participants who exhibited positive symptomatology and at least one diagnostic sign were eligible for inclusion in the study. This two-step diagnostic approach was conducted in accordance with the recommendations of the TFOS DEWS II Diagnostic Methodology Subcommittee, ensuring that all enrolled subjects met standardized diagnostic criteria DED [6,7]:

- Participants completed the OSDI questionnaire via a self-administered online form to assess the presence of symptomatology [26]. Positive symptomatology is associated with an OSDI value ≥ 13 points.
- Tear film osmolarity was measured using a TearLab osmometer (TearLab, San Diego, CA, USA) [27]. The diagnostic cut-off values for DED diagnosis were a tear osmolarity ≥ 308 mOsm/L.
- BUT and corneal staining were recorded using a Topcon^®^ SL-D4 slit-lamp equipped with a DV-3 video camera (Topcon Corporation, Tokyo, Japan) and non-preserved fluorescein [12]. The diagnostic cut-off values for DED diagnosis were a BUT ≤ 10 s and/or corneal staining (Oxford grade) ≥ 2.

Participants who fulfilled both criteria (positive symptoms and at least one positive sign) were diagnosed with DED and included in the study. Subsequently, these participants were classified into subtypes based on their TMH, measured under slit-lamp illumination. Those with TMH < 0.20 mm were categorized as having ADDE, while those with TMH ≥ 0.20 mm were classified as Non-ADDE [6,7]. Table 1 summarizes the diagnostic procedure following the TFOS DESWS II Diagnostic Methodology Subcommittee recommendations [6,7].

### 2.3. TMCA Acquisition and Evaluation

#### 2.3.1. Image Acquisition and Selection

In preparation for subsequent analysis, all recordings were conducted under fluorescein instillation, as images lacking fluorescein lacked sufficient contrast for the computer-assisted image semi-automatic analysis protocol proposed in this study. Prior to tear film recording, a 2 µL volume of non-preserved 2% sodium fluorescein was instilled in the inferior temporal bulbar conjunctiva using a micropipette [28]. Within 30 s of fluorescein instillation, subjects assumed a chin rest position, fixating on a target to maintain primary eye gaze. The lower tear meniscus was observed using a Topcon SL-D4 biomicroscope set at 40×, and videos were recorded with a Topcon DV-3 digital camera attached to the biomicroscope. Videos were stored at a spatial resolution of 1024 × 768 pixels in the RGB color space on a connected computer via the software Topcon IMAGEnet i-base version 3.22.0 (Topcon Corporation, Tokyo, Japan) provided by the manufacturer. After recording, the subject blinked naturally three or four times to distribute fluorescein evenly over the cornea [28]. To minimize reflex tearing due to direct light into the pupil, a short constant cobalt blue light beam of 3 mm wide and 5 mm high with moderate illumination and specific filters was used [21]. A cobalt blue light and a Wratten 12 yellow filter were also used to enhance tear film visibility. The video capture settings were standardized, focusing on the central meniscus at the 6 o’clock position below the pupil center [14]. Always obtaining the same image position was crucial for the subsequent image analysis by the software. Decentered or improperly focused images could not be adequately analyzed by the program.

From each recorded video, a masked observer extracted one image of the lower tear meniscus for further analysis. The selection criteria ensured stability and minimal changes in the meniscus, captured 2–3 s after blinking [3,14,29].

#### 2.3.2. Semi-Automatic Evaluation of the TMCA Parameter

The computer-assisted image analysis was carried out using ImageJ software v1.53d, an open-source Java-based image processing tool provided by the National Institutes of Health in Bethesda, MD, USA (https://imagej.net/ij/, accessed on 25 March 2025) [19,21]. Prior to this study, the authors used ImageJ to establish that one millimeter on a ruler captured by the current DV-3 digital camera attached to the Topcon SL-D4 slit-lamp at 40× magnification corresponds to a digital length of 300 pixels. These pre-study calibration data were then applied to convert ImageJ pixel measurements into estimated areas in mm^2^ for subsequent statistical and data analysis. To do this in ImageJ software, click on “Analyze >> Set Scale” and then set “Distance in pixels” as 300 and “Unit of length” as millimeters. This will set a proportion of 300 pixels per millimeter, allowing for accurate area measurements.

**Figure 1:**
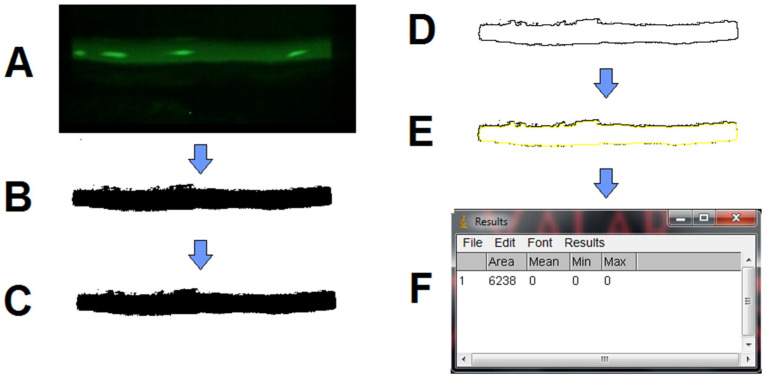
Measurement example of TMCA with ImageJ step by step. (**A**) Extracted image; (**B**) Process >> Binary >> Make Binary; (**C**) Process >> Binary >> Fill Holes; (**D**) Process >> Binary >> Make Outline; (**E**) >> Wand tool; (**F**) Analyse >> Measure.

Images obtained from the tear meniscus, recorded after fluorescein instillation, were imported into ImageJ software (Figure 1A). Subsequently, the image underwent transformation through the “Process >> Binary >> Make Binary” command, converting the green-tinted meniscus into a black shape and the remaining image (the black part) into a white background (Figure 1B). To address any potential “holes” resulting from this transformation, the “Process >> Binary >> Fill Holes” command was applied (Figure 1C). The external profile of the resulting black shape was then isolated using the “Process >> Binary >> Make Outline” command (Figure 1D). Finally, the generated profile was selected with the “Wand tool” (Figure 1E), and the area size was calculated using the “Analyse >> Measure command”, providing the results in a separate window (Figure 1F). The TMCA data from each participant was collected in a Microsoft Excel spreadsheet (Microsoft, Washington, DC, USA) for management and subsequent statistical analysis.

### 2.4. Statistical Analysis

SPSS statistical software version 25.0 for Windows (SPSS Inc., Chicago, IL, USA) was employed to conduct the data analysis. The significance level was set at *p* ≤ 0.05 for all statistical analyses. Participants were categorized according to their diagnosis using TFOS DEWS II criteria and were assigned to their respective groups [7,12].

Correlations between refractive parameters TFOS DEWS II criteria and the TMCA were assessed by Spearman’s correlation test. Correlations were classified as weak (0.20 to 0.40), moderate (0.41 to 0.60), good (0.61 to 0.80), or strong (0.81 to 1.00) [30]

To identify the optimal cut-off value distinguishing between Non-ADDE and ADDE diagnoses, both subjective and objective evaluations were employed, and Receiver Operating Characteristics (ROCs) procedures were applied [31]. Sensitivity and specificity were computed for various cut-off values, and a graphical representation illustrating the relationship between sensitivity and (1-specificity) was generated. The optimal cut-off value, selected as the hinge point of the curve, offered the best combination of sensitivity and specificity. The discriminatory power of the predictive classification model was assessed using the Area Under the Curve (AUC) value, ranging from 0 to 1, with higher values indicating superior predictive ability. Additionally, upper and lower 95% Confidence Intervals (CIs) for the AUC were provided. The maximum value of Youden’s J statistic determined the best cut-off criterion based on each ROC curve.

In a subsequent reanalysis, cross-validation was conducted on parameters exhibiting significant diagnostic capability to validate the identified cut-off. The variables were transformed into dichotomous parameters based on the calculated cut-off, and their association with the initial diagnosis was examined using Cramer’s V statistic. Additionally, a confusion matrix was generated by cross tabulating the classifications obtained from the TMCA cut-off against the reference standard provided by the TFOS DEWS II criteria. Based on this matrix, diagnostic agreement metrics were calculated, including the proportions of false positives (participants classified as ADDE by the TMCA cut-off but as Non-ADDE by the TFOS criteria) and false negatives (participants classified as Non-ADDE by the TMCA cut-off but as ADDE by the TFOS criteria). Furthermore, standard diagnostic performance measures such as accuracy (the proportion of correctly classified cases), false positive rate, and false negative rate were derived from the matrix to provide complementary insight into the practical diagnostic utility of the proposed threshold.

## 3. Results

Table 2 shows the descriptive statistics of the study sample, stratified into two groups: participants Non-ADDE (*n* = 74) and those diagnosed with ADDE (*n* = 46).

Table 3 shows the correlation between the TMCA and the diagnostic and classification parameters used in the study. A statistically significant positive correlation was found between TMCA and TMH (Spearman’s rho correlation, r = 0.417, *p* < 0.001), whereas no significant correlations were found between TMCA and other parameters (all *p* ≥ 0.087).

**Figure 2:**
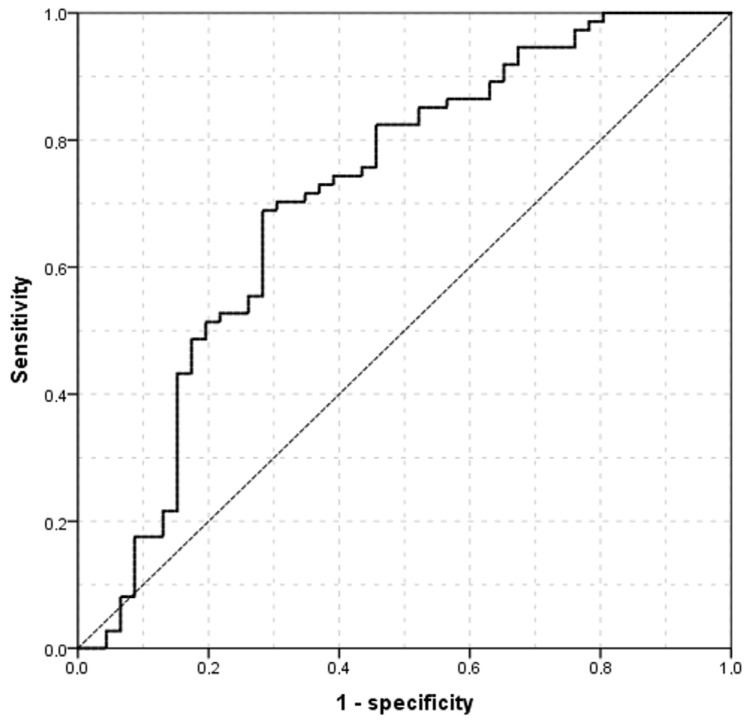
ROC curve showing the relationship between sensitivity and specificity of the TMCA (Non-ADDE vs. ADDE) according to theoretical thresholds; for each of the values observed in the study population (from the lowest to the highest in either Non-ADDE or the ADDE group). The sensitivity and specificity indexes have been calculated and reported in the graph. TMCA = Tear Meniscus Central Area. ADDE: Aqueous Deficient Dry Eye. Non-ADDE: Non-Aqueous Deficient Dry Eye.

ROCs procedures showed that objective semi-automatic TMCA evaluation has a significant diagnostic capability to differentiate between participants’ subtypes (AUC = 0.714 ± 0.051, *p* < 0.001, 95% CI = 0.613–0.814, Figure 2). By calculating the Youden’s index (Youden’s J statistic = 0.407), a cut-off value for differentiate between Non-ADDE and ADDE participants of 54.62 mm^2^ (specificity: 71.7%; sensitivity: 68.9%) (Figure 2) was found. The AUC shown in Figure 2 graphically represents the ability of the current model (objective semi-automatic TMCA evaluation) to distinguish between participants with ADDE and those without (Non-ADDE), thereby supporting the potential of the proposed technique to measure TMCA as an indirect indicator of tear volume.

To further explore the diagnostic performance of the proposed TMCA cut-off value, a cross-validation analysis was performed on a random subsample comprising 80% of the total dataset. As shown in Table 4, a significant association was found between the TMCA-based classification and the reference standard proposed by the TFOS DEWS II Diagnostic Methodology Subcommittee (Cramer’s V = 0.410, *p* < 0.001), supporting the discriminative ability of the method to distinguish between Non-ADDE and ADDE cases.

Additionally, the cross-tabulation allowed for the calculation of diagnostic agreement metrics (Table 4): the overall accuracy was 70.8%, with a false positive rate of 38.1% and a false negative rate of 22.2%. These values highlight a tendency of the TMCA-based classification to overestimate ADDE in some cases, although the general agreement remains clinically relevant.

## 4. Discussion

The present study makes a valuable contribution to the understanding of DED diagnosis by emphasizing the significance of the tear meniscus, particularly in the context of ADDE, as a reliable estimation of tear volume. The use of a semi-automatic method for TMCA measurement, facilitated by ImageJ software, addresses the limitations posed by expensive and specialized imaging equipment [16]. The establishment of a diagnostic cut-off value for TMCA provides a practical tool for clinicians to differentiate between Non-ADDE and ADDE participants, aiding in accurate diagnosis and subsequent management. Therefore, with a simple image of the central meniscus captured using a slit-lamp after fluorescein instillation, the TMCA can be measured, making the current procedure a reliable discriminator between individuals with ADDE and those without. It is essential to note that clinicians routinely use fluorescein staining during daily eye examinations. Previous studies have underscored the importance of parameters such as TMH or the radius of curvature in DED diagnosis, implicating their relevance in evaluating ocular health [2,8,13,29,32,33,34,35,36]. In recent decades, various non-invasive methods for measuring TMH have been proposed, in addition to the traditional measurement with a slit-lamp [37]. However, these proposed methods have certain drawbacks, such as excessive cost and large instrument size [10]. The inclusion of new parameters such as the TMCA measurements may complement current diagnostic algorithms recommended by TFOS DEWS II, particularly in cases where tear volume estimation is critical for subtype differentiation. Despite its potential to enhance diagnostic accuracy and streamline clinical workflows, the implementation of the present proposed tool in routine practice presents several practical hurdles. First, the cost of integrating image capture and processing infrastructure may be a limiting factor, particularly in under-resourced settings. Second, clinicians and technicians would require targeted training to ensure proper image acquisition and interpretation of algorithm outputs. Finally, the compatibility of the system with existing slit-lamp hardware, especially older models, needs to be considered, and standardized mounting or digitalization protocols may be required for widespread adoption. However, incorporating this parameter could aid clinicians in achieving a more comprehensive evaluation of DED severity and subtype classification, ultimately contributing to better-tailored therapeutic strategies. Although the present study was designed according to the TFOS DEWS II diagnostic framework [4], the recent publication of TFOS DEWS III introduces a refined, though largely consistent, update to the classification and diagnostic approach for DED [5]. Future research should explore the applicability of the proposed TMCA-based method within this updated diagnostic context.

Several studies have reported mean TMH values across different cohorts of participants with DED, highlighting variations based on the underlying etiology. For instance, TMH values have been reported to range from 0.19 ± 0.05 mm to 0.20 ± 0.04 mm in participants diagnosed with ADDE, from 0.271 ± 0.117 mm to 0.29 ± 0.09 mm in those with meibomian gland dysfunction (MGD), from 0.19 ± 0.05 mm to 0.20 ± 0.04 mm in participants with combined ADDE and MGD, and approximately 0.169 ± 0.029 mm in participants with DED of unspecified origin. These findings suggest that TMH values may serve as a potential biomarker for differentiating DED subtypes and guiding clinical management [38,39,40]. Also, the current research group investigated the importance of TMH in distinguishing between DED subtypes, such as ADDE, Mixed DED and Evaporative Dry Eye (EDE), by implementing ROC curve analysis. A previous report demonstrated the ability to discriminate between ADDE and EDE by establishing a TMH cut-off value of 0.159 mm, when measured under Tearscope illumination [12]. Nevertheless, research specifically investigating meniscus area parameters as a diagnostic criterion has been limited. This gap in the literature highlights the need for more comprehensive studies to evaluate the diagnostic utility of meniscus area measurements, which could provide a more nuanced understanding of tear film dynamics and contribute to the development of more robust diagnostic tools for DED.

Previous reports have measured tear meniscus area parameters measured via OCT, such as Tear Meniscus Cross-Sectional Area (TMA) in both the upper and lower (LTMA) meniscus [8,41,42]. These parameters, particularly LTMA, have shown significant reductions in dry eye patients compared to controls, revealing a statistical correlation with key clinical indicators such as BUT, corneal and conjunctival staining, gender, age, and symptoms [41]. Previous studies have suggested the possibility of calculating the total tear film volume from cross-sectional TMA measured in a specific eyelid length area of only 1 mm [42]. The limited literature available on the tear meniscus area highlights the need for further research in a field that could contribute to faster and more accurate procedures for the diagnosis and management of DED, by enabling proper classification of its subtypes and facilitating more personalized treatments to improve the quality of life of those affected. Although the semi-automated evaluation of the TMCA demonstrated a statistically significant ability to distinguish between ADDE and Non-ADDE participants in the present sample (AUC = 0.714), its diagnostic performance should be interpreted in the context of previously validated tear meniscus parameters. For instance, OCT-derived TMH has shown a superior diagnostic power in mild DED or rosacea cases (AUC = from 0.628 to 0.978) compared to both TMA (AUC = from 0.666 to 0.960) and TMCA in the present study [11,43], while inter-eye differences in inter-eye absolute difference in TMH (TMH|OD–OS|) have also demonstrated relevant discriminative capability (AUC = 0.719) [44]. Furthermore, values obtained via Tearscope-assisted TMH assessments have reported AUCs of 0.843 and 0.953 for differentiating ADDE from EDE and stratifying ADDE severity, respectively [12]. Compared with these methods, TMCA presents a slightly lower but still acceptable discriminative performance, suggesting that while it may not surpass established parameters in terms of diagnostic accuracy, it could provide complementary insights, particularly when integrated into multimodal dry eye evaluation strategies.

One of the main strengths of the present study was the large sample employed, which provided a statistical significance that may allow generalizability of the results to a broader population of individuals diagnosed with ADDE. While the sample included a relatively balanced number of participants with and without ADDE, it consisted primarily of young adults and showed a predominance of female participants, which may restrict the applicability of the findings to older or more diverse populations. Furthermore, most participants with ADDE presented mild to moderate signs and symptoms, with relatively few cases of severe disease, which may also limit the extrapolation of the results to more advanced stages of the condition [12,44]. In addition, this technique can be implemented in a reliable manner in clinical offices since it is not costly for many practitioners, a slit-lamp is always available, and the ImageJ software is open-source. However, it is essential to acknowledge potential limitations, such as the need for further research to explore the applicability of the semi-automatic TMCA measurement method in diverse clinical settings, such as different slit-lamps or cameras to video capture. Another potential limitation is the inherent variability that may arise from semi-automated image segmentation, particularly when operated by different clinicians or under varying image acquisition conditions. Standardized protocols for image acquisition and processing would be essential to minimize this variability. Additionally, longitudinal studies could provide insights into the dynamic changes in TMCA over time and its correlation with the progression of ADDE [45]. The use of fluorescein may represent a relevant source of variability in TMCA measurement. While this dye enhances image contrast and facilitates the visualization of the tear meniscus, its instillation can alter the natural morphology and properties of the tear film, potentially affecting the accuracy of the results. Several studies have reported that fluorescein may increase TMH due to its impact on tear film stability. For instance, Lim and Lee observed that TMH values measured five minutes after instillation of a 0.25% fluorescein solution were comparable to those obtained without fluorescein, suggesting that timing is a critical factor to ensure reliability [46]. Although fluorescein-based measurements are widely used in clinical settings and often considered to have minimal influence on TMH values [47], the possibility that it may modify tear film dynamics should not be overlooked when interpreting TMCA. Moreover, accurate and consistent fluorescein distribution depends on individual blinking patterns and tear kinetics, which may vary significantly across subjects and introduce additional variability. In this study, a standardized protocol was implemented to mitigate such effects, including the acquisition of images within a specific time window shortly after blinking, once fluorescein distribution was presumed stable [48]. Even so, further investigations are needed to define optimal protocols regarding dye concentration, instillation timing, and image acquisition parameters in order to enhance comparability and reproducibility across future studies.

Although the algorithm showed promising results in the present study dataset, its performance may be influenced by artifacts such as poor illumination, tear film disturbances, or lid interference, which can compromise image quality. Future work should evaluate its robustness against these common sources of noise. Additionally, inter-device variability, arising from differences in camera resolution, lighting conditions, or optical alignments, may affect reproducibility and generalizability across different clinical settings. Calibration procedures and domain adaptation strategies may be necessary to ensure consistent performance across various slit-lamp platforms. Another potential methodological limitation of the proposed image analysis protocol is that it relies on having a clearly isolated region of interest containing only the tear meniscus prior to binarization. In the present study, this condition was fulfilled, as the captured images already included only the meniscus area, making the use of threshold adjustment unnecessary. However, in less controlled image acquisitions, applying an appropriate threshold (via Image >> Adjust >> Threshold in ImageJ) prior to binarization may be essential to ensure that only relevant structures are retained in the binary image. Future implementations of this technique should consider this step when working with more complex or cluttered images.

Future research in the field of tear meniscus assessment should concentrate on evaluating the variability among different imaging devices. These include slit-lamp integrated cameras, smartphone adaptable systems that attach to slit-lamp oculars, and multi-diagnostic platforms capable of capturing cobalt blue light images that enhance fluorescein-stained tear meniscus visibility. The advancement of research in the field of the tear meniscus area has the potential to greatly improve our understanding of DED and its impact on ocular structures.

## 5. Conclusions

In conclusion, the present study successfully validates a semi-automatic method for measuring TMCA as a valuable tool in differentiating between Non-ADDE and ADDE participants. The establishment of a practical cut-off value and the confirmation through cross-validation enhance the method’s applicability in clinical settings; however, further validation in independent cohorts is necessary to confirm its generalizability.

## Figures and Tables

**Table 1:** Summary of diagnostic criteria and classification of DED and ADDE.

Interpretation		OSDI	Tear Film Osmolarity (mOsm/L)	BUT (s)	Corneal Staining (Oxford Grade)	TMH (mm)
Diagnosis	Categorization					
Non-DED	-	<13	<308	>10	<2	-
DED	Non-ADDE	≥13	≥308 (or)	≤10 (or)	≥2 (or)	≥0.20
DED	ADDE	≥13	≥308 (or)	≤10 (or)	≥2 (or)	<0.20

OSDI = Ocular Surface Disease Index. BUT = Tear Film Break-Up Time. TMH = Tear Meniscus Height. DED = Dry Eye Disease. ADDE = Aqueous Deficient Dry Eye.

**Table 2:** Descriptive statistics of the sample.

Characteristic	Value	
	Non-ADDE *n* = 74	ADDE *n* = 46
Female gender (%)	48 (64.9%)	36 (78.3%)
Age (years), mean (SD)	21.37 (4.88)	22.76 (7.98)
OSDI, median (IQR)	16.67 (14.58–17.19)	16.67 (14.58–22.76)
BUT (s), median (IQR)	8.10 (5.21–9.77)	6.12 (4.29–8.74)
Corneal staining (Oxford grade), median (IQR)	0.00 (0.00–0.25)	0.00 (0.00–1.00)
Osmolarity (mOsm/L), mean (SD)	308.53 (14.58)	312.30 (12.63)
TMH (mm), mean (SD)	0.237 (0.03)	0.154 (0.03)
TMCA (mm^2^), mean (SD)	62.23 (15.60)	54.94 (29.46)

SD = Standard Deviation. IQR = Interquartile Range. OSDI = Ocular Surface Disease Index. BUT = Break-up Time. TMH = Tear Meniscus Height. TMCA = Tear Meniscus Central Area. ADDE = Aqueous Deficient Dry Eye.

**Table 3:** Correlation between the TMCA and the diagnostic and classification parameters used in the study.

		OSDI	Tear Film Osmolarity (mOsm/L)	BUT (s)	Corneal Staining (Oxford Grade)	TMH (mm)
TMCA (mm^2^)	r	−0.091	0.157	−0.070	−0.094	0.417
	*p*	0.320	0.087	0.445	0.308	<0.001

OSDI = Ocular Surface Disease Index. BUT = Break-up Time. TMH = Tear Meniscus Height. TMCA = Tear Meniscus Central Area. ADDE = Aqueous Deficient Dry Eye.

**Table 4:** Cross-tabulation between TMCA-based classification and TFOS DEWS II diagnostic categories.

		TFOS DEWS II Diagnostic Methodology Subcommittee		
		Non-ADDE	ADDE	Total
**TMCA** **cut-off value**	**Non-ADDE**	26 (27.1%)	16 (16.7%)	42 (43.8%)
	**ADDE**	12 (12.5%)	42 (43.8%)	54 (56.3%)
	**Total**	38 (39.6%)	58 (60.4%)	96 (100%)

TFOS DEWS II = Tear Film Ocular Surface second Dry Eye Workshop. TMCA = Tear Meniscus Central Area. ADDE = Aqueous Deficient Dry Eye.

## Data Availability

The original contributions presented in this study are included in the article. Further inquiries can be directed to the corresponding author.
